# Study protocol for an evaluation of the effectiveness of ‘care bundles’ as a means of improving hospital care and reducing hospital readmission for patients with chronic obstructive pulmonary disease (COPD)

**DOI:** 10.1186/s12890-016-0197-1

**Published:** 2016-02-25

**Authors:** M. J. E. Chalder, C. L. Wright, K. J. P. Morton, P. Dixon, A. R. Daykin, S. Jenkins, J. Benger, J. Calvert, A. Shaw, C. Metcalfe, W. Hollingworth, S. Purdy

**Affiliations:** School of Social and Community Medicine, University of Bristol, Bristol, UK; Sue Jenkins Consulting, Taunton, UK; Faculty of Health and Applied Sciences, University of the West of England, Bristol, UK; Southmead Hospital, North Bristol NHS Trust, Bristol, UK; Bristol Randomised Trials Collaboration, University of Bristol, Bristol, UK

**Keywords:** COPD, Care bundles, Delivery of care, Quality improvement, Admission avoidance

## Abstract

**Background:**

Chronic Obstructive Pulmonary Disease is one of the commonest respiratory diseases in the United Kingdom, accounting for 10 % of unplanned hospital admissions each year. Nearly a third of these admitted patients are re-admitted to hospital within 28 days of discharge. Whilst there is a move within the NHS to ensure that people with long-term conditions receive more co-ordinated care, there is little research evidence to support an optimum approach to this in COPD. This study aims to evaluate the effectiveness of introducing standardised packages of care i.e. care bundles, for patients with acute exacerbations of COPD as a means of improving hospital care and reducing re-admissions.

**Methods / Design:**

This mixed-methods evaluation will use a controlled before-and-after design to examine the effect of, and costs associated with, implementing care bundles for patients admitted to hospital with an acute exacerbation of COPD, compared with usual care. It will quantitatively measure a range of patient and organisational outcomes for two groups of hospitals - those who deliver care using COPD care bundles, and those who deliver care without the use of COPD care bundles. These care bundles may be provided for patients with COPD following admission, prior to discharge or at both points in the care pathway. The primary outcome will be re-admission to hospital within 28 days of discharge, although the study will additionally investigate a number of secondary outcomes including length of stay, total bed days, in-hospital mortality, costs of care and patient / carer experience. A series of nested qualitative case studies will explore in detail the context and process of care as well as the impact of COPD bundles on staff, patients and carers.

**Discussion:**

The results of the study will provide information about the effectiveness of care bundles as a way of managing in-hospital care for patients with an acute exacerbation of COPD. Given the number of unplanned hospital admissions for this patient group and their rate of subsequent re-admission, it is hoped that this evaluation will make a timely contribution to the evidence on care provision, to the benefit of patients, clinicians, managers and policy-makers.

**Trial registration:**

International Standard Randomised Controlled Trials – ISRCTN13022442 - 11 February 2015

## Background

### Epidemiology of COPD

Chronic Obstructive Pulmonary Disease (COPD) is one of the most common respiratory diseases in the United Kingdom [1] (UK). It is estimated that the prevalence of COPD in the UK is over 3 million, of which only about 900,000 cases have been diagnosed [2]. The majority of people with COPD also have other medical problems; most commonly ischaemic heart disease which occurs in some 25 % of patients [3]. Many people discharged from hospital after an acute exacerbation of chronic obstructive pulmonary disease (AECOPD) also report feelings of depression (64 %) and anxiety (40 %). This multi-morbidity means that managing patients’ healthcare needs is challenging [4–6].

### COPD and unplanned hospital admissions

COPD accounts for 10 % of unplanned hospital medical admissions – totalling over 90,000 annually in the UK [1]. Nearly a third of these patients are re-admitted to hospital within 28 days of discharge [7] and this proportion is steadily rising, with a 2 % increase in the re-admission rate between 2003 and 2008 [3]. During the same time period, there has been little change in in-hospital mortality rates; estimated at 7.5 % in 2003 and 7.7 % in 2008 [7]. As well as being an important cause of unplanned admissions, COPD is the second most common cause of emergency admission to hospital [2] and the fifth largest cause of re-admission [8], costing the National Health Service (NHS) an estimated £491 million per year [9]. Indeed, the number of admissions has increased by 50 % in the last decade and now accounts for one million bed days per annum [10]. All of these figures suggest that acute, urgent and emergency COPD healthcare will continue to challenge the NHS for the foreseeable future and, as such, there is considerable pressure on managers and clinicians to work to resolve the issue.

### Evidence-based COPD care

Emergency admissions to hospital for long-term conditions - including COPD - form part of the NHS Outcomes Framework [11] and are the subject of a number of admission reduction initiatives [12-14]. It has been suggested that 10–34 % of COPD admissions could be avoided through the implementation of evidence-based care [7, 11]. A Royal College of Physicians Audit conducted in 2003 [15] found that, on average, patients spend 8.7 days in hospital during an admission for COPD but also that there was wide variation in terms of both treatment provision and outcomes amongst hospitals. This disparity was particularly marked in relation to mortality. The audit showed that a significant proportion of the observed variability could be explained by availability and access to expert care and evidence-based interventions which have, in turn, resulted in a reduced length of stay and lower mortality [7]. This, therefore, presents a potential opportunity to improve outcomes for patients with COPD [3] by ensuring that their care is consistently provided to a high standard.

### COPD care bundles

One example of an evidence-based intervention is the use of care bundles. These are a simple way of focusing service improvement efforts on a set of defined actions which will contribute to the achievement of a clearly specified aim [10]. Improvement theory suggests that, properly implemented, the use of care bundles should enable clinical teams to concentrate on a range of measurable activities and optimise certain associated outcomes [10]. In practical terms, this should mean that protocol-based care bundles for COPD will enable staff to see quickly what course of action should be taken, when and by whom, and that this will result in standardisation of practice in the treatment of patients. COPD care bundles could also be an important tool in improving the quality of care, since any deviation from the agreed care pathway can be measured easily, enabling systemic factors that might inhibit provision of best care to be identified and subsequently addressed.

### Justification for research

Some evaluation of the effectiveness of a number of types of care bundles [10, 16] has taken place, but there is little UK-based evidence at present^,^ about their impact on the processes and outcomes of care for COPD [17, 18]. The preliminary findings of single pilot sites in the UK indicate that the implementation of in-patient care pathways or bundles can improve clinical outcomes such as mortality, hospital re-admission rates and length of hospital stay. Indeed, a study by Hopkinson and colleagues has shown a downward trend in 30-day re-admissions in patients with COPD in whom a bundle approach to discharge was applied [19]. However, in order to improve outcomes for COPD, greater understanding of clinical care, service delivery gains and cost-effectiveness is required. In doing so, more in-depth knowledge will be gained about the potential for COPD care bundles to reducing unplanned hospital admissions and improve patient outcomes.

### Aim

This study aims to evaluate the effectiveness of introducing standardised packages of care i.e. bundles, for patients with an acute exacerbation of COPD as a means of improving hospital care and reducing re-admissions.

## Methods

### Study design

**Fig. 1:**
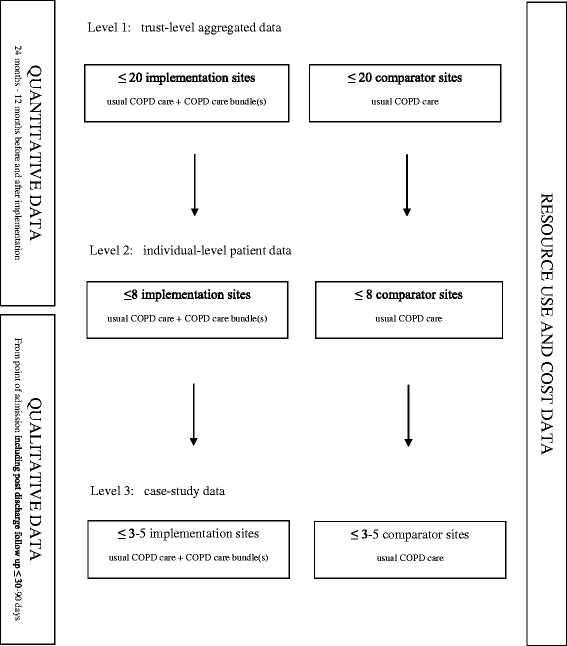
Schematic representation of study design

The research will use a controlled before-and-after design with nested case studies to compare the outcomes of care following the introduction of care bundles with usual care for patients admitted to hospital with AECOPD. Study sites will participate in up to three different levels of data collection and these are described in the schematic presented in Fig. 1.

### Study setting

The evaluation will be conducted in up to 40 acute hospitals within England and Wales. The aim is to include a group of hospitals who offer care to patients admitted with COPD using a care bundle approach (i.e. implementation sites) and a broadly comparable group of hospitals who deliver care for the same patient population without the use of care bundles (i.e. comparator sites).

### Inclusion and exclusion criteria

The target population for Level 1 and Level 2 participation will be acute hospitals with an emergency department (ED) and adult respiratory in-patient care. Hospitals outside England and Wales will be excluded. The target population for Level 3 participation will be people over the age of 18, admitted to hospitals participating in Level 1 or Level 2 data collection where their primary cause of admission is COPD as defined by the International Statistical Classification of Diseases and Related Health Problems [20] (ICD-10) using diagnostic codes J41-J44, or carers of such individuals. This may be a first, second or indeed a subsequent admission for that patient during the study period, but excludes admissions related to any form of elective treatment for COPD.

### Recruitment

#### Identification of sites

There are two types of participating sites in the study – implementation sites and comparator sites – and both will be identified using similar methods. In order to maximise the number and diversity of hospitals given the opportunity to participate in the research, a range of approaches will be used. These include:

- advertising calls for interest on the British Thoracic Society (BTS) website (https://www.brit-thoracic.org.uk/)
- advertising calls for interest on the respiratory section of the National Institute of Health Research (NIHR) Clinical Research Network (CRN) website (https://www.crn.nihr.ac.uk/news/call-for-hospitals-not-currently-using-care-bundles-for-copd-patients/)
- approaching respiratory specialists from NHS trusts at BTS scientific meetings
- communicating calls for interest via NIHR CRN-nominated local respiratory research leads
- communicating calls for interest via NIHR CRN delivery managers
- generating new clinical contacts from known ones using ‘snowballing’ techniques
- making ‘cold calls’ to major acute hospitals not otherwise contacted

#### Recruitment of sites

Once a hospital has expressed their interest in taking part in the research, they will be sent further information about the study including a link to the NIHR CRN Portfolio database (http://public.ukcrn.org.uk/Search/StudyDetail.aspx?StudyID=17828), a link to the study website hosted by the University of Bristol (http://www.bristol.ac.uk/primaryhealthcare/researchthemes/copd/), a research summary, the full study protocol and a copy of the BTS COPD care bundles pilot study report [10]. Next, the hospital’s status as either an implementer of COPD care bundles or a comparator delivering standard care will be determined, and the site asked to sign a formal agreement to be a participate in the evaluation accordingly. Following this, a member of the research team will submit the relevant site-specific information (SSI) via the Integrated Research Application System (IRAS) website (www.myresearchproject.org.uk/) and liaise directly with the site’s Research & Development (R&D) team to ensure all appropriate permissions and approvals are in place. This will include the appointment of a local Principal Investigator (PI) at each site to take responsibility for data collection and patient care.

#### Allocation to study level and matching of sites

Eight implementation sites will be assigned to Level 2 for data collection and analysis purposes. This allocation will depend on two conditions being met – namely, delivering both an admission and a discharge bundle for COPD care and being willing to report on the Level 2 data requirements. Any remaining implementation sites will be allocated to Level 1 participation. Eight further sites will be selected from amongst the comparator sites able to fulfil the same Level 2 conditions in order to obtain eight matched implementation-comparator site pairs. The pairs will be matched as closely as possible on the following pre-specified criteria: number of COPD admissions, 28-day re-admission rates and COPD mortality rates.

#### Identification of participants

Up to ten individuals (i.e. patients who have been admitted following an acute exacerbation of COPD, or their carers) will be identified by respiratory team members as being appropriate for participation in the study at a sample of sites chosen as Level 3 case study locations. Individuals will be selected at various stages of the COPD patient care pathway, including the emergency department, admissions units, and in-patients wards. This assessment will take account of their health status, level of cognition and ability to communicate easily with the research team.

#### Recruitment of participants

Patients and their carers will be invited to take part in the study following the procedures set out in the International Conference on Harmonisation (ICH) good clinical practice (GCP) guidelines (www.ich.org/), with informed written consent being obtained prior to any data collection. Where appropriate, individuals will be interviewed during the period of admission, as well as being interviewed at 30 and 90 days post-discharge, either face-to-face or via telephone. Staff involved in an individual patient’s care will also be invited to interview in a similar manner and at similar time-points.

### Intervention / implementation

**Fig. 2:**
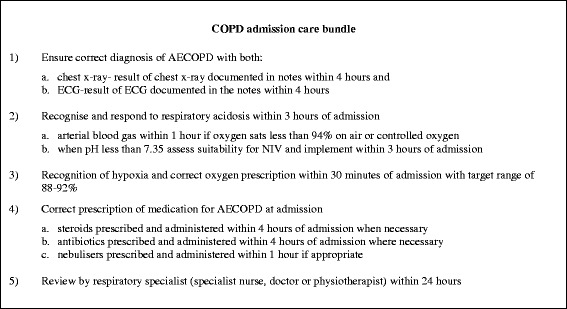
Summary of COPD admission care bundle

**Fig. 3:**
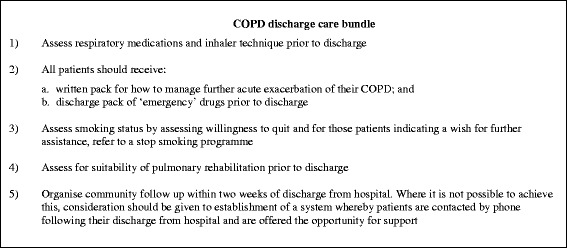
Summary of COPD discharge care bundle

The intervention of interest in this study is COPD care bundles delivered as part of in-hospital patient care. In order to encompass the full range of measures required, two separate care bundles were derived [10]. The first is to be completed at the point of hospital admission and aims to reduce length of stay and in-hospital mortality for COPD. The second is to be completed before discharge from hospital and aims to reduce re‐admissions for COPD. Together, these two co-ordinated packages of care comprise ten evidence-based actions which, when successfully completed, are designed to lead to an improvement in the overall care for those patients admitted to hospital with an acute exacerbation of chronic obstructive pulmonary disease. For the purposes of this study, all participating hospitals who deliver some form of care bundles i.e. implementation sites at either Level 1 or 2, will be offered a series of training and networking opportunities in order to promote quality improvement and to facilitate their local implementation processes. Further details of both care bundles are provided in Figs. 2 and 3.

### Outcomes and outcome measures

The primary outcome of the evaluation is COPD re-admission rate at 28 days, which is the proportion of people re-admitted to hospital within 28 days of discharge for an AECOPD. This will be measured using Level 1 data from each of the participating sites. We will calculate the mean change in 28-day re-admission rate following the introduction of care bundles for each site, and then compare these site level summaries between implementation and comparator arms.

A variety of secondary outcome data will be collected over the course of the evaluation, including:

- total number of COPD admissions
- COPD admission rate
- in-hospital mortality for COPD admissions
- length of stay for COPD admissions
- total bed days for COPD admission
- COPD re-admission rate at 90 days
- overall re-admission rate at 28 days
- total number of patients with COPD seen / discharged from the emergency department
- total number of patients with COPD in whom bundle used – at implementation sites only
- cost-effectiveness of COPD care bundles from an NHS perspective
- context and process of care
- impact of the care bundles on patients, carers and staff – at implementation sites only

### Data collection

Data will be collected at each implementation site over a minimum 24-month period – 12 months immediately preceding the implementation of the COPD care bundle(s) and 12 months after the start of implementation. Since it is likely that some sites will have an implementation phase during which their COPD care bundle(s) become embedded into clinical practice, data will also be collected during this time-period as appropriate. Whatever the total data collection period at implementation sites, data collected by comparator sites will reflect a similar distribution of 12-month ‘before’ and 12-month ‘after’ calendar time-period. One implementation site will be recruited to pilot data collection at all three levels.

Sites will appoint appropriate people to report their data to the research team. The frequency of the data extraction will depend on the time-period that the site is reporting for, as well as the resources available to them. For example, if all of the data extraction is retrospective, the hospital may choose to provide all the data in one tranche. If however, the data extraction is conducted in ‘real time’, they may choose to provide it on a monthly basis. The data will be compiled and checked for validity and consistency by a member of the research team.

#### Level 1 data collection

All sites will report a range of aggregated routine data including COPD admission rate, COPD re-admission rate at 28 and 90 days, overall re-admission rate at 28 days, in‐hospital mortality for COPD admissions, length of stay for COPD admissions, total bed days for COPD admissions, total number of COPD patients seen and discharged by ED and – at implementation sites - the total number of patients in whom the bundle was used.

#### Level 2 data collection

In addition to the Level 1 data, Level 2 sites will be required to provide more detailed, pseudo-anonymised individual patient-level data including age, sex, ethnicity and geographical variables. This form of data collection will also capture non-identifiable clinical information including admission month and year; source of admission; ICD-10 diagnosis codes; Office of Population, Census and Surveys (OPCS) procedure codes; length of stay - total and by ward type; discharge destination; healthcare resource group (HRG) codes; pseudo-anonymised consultant and GP practice codes; re-admission at 28 days for COPD; re-admission at 28 days for all cause admissions; re-admission at 90 days for COPD; out-patients appointments; ED appointments; in‐hospital mortality; 90-day mortality including number of days after discharge that death occurred by data linkage to death registry information and – at implementation sites – the total number of patients in whom the bundle was used. Level 2 sites will also report on process measures associated with the delivery of components of COPD care by returning information extracted from a randomly selected sample of 140 patient records per site.

#### Level 3 data collection

In addition to the Level 1 and 2 data, a selection of sites will form Level 3 case studies and be examined in detail as regards the process of care bundle implementation, the context in which the care bundles are delivered, the impact of the care bundles on staff, patients and carers, and – where more appropriate – the nature of usual COPD care. Data collection at this level will be carried out through non-participant observation and in-depth interviewing, as well as document analysis. It will be supported by the use of topic guides and observation schedules, and conducted by an experienced qualitative researcher at both implementation and comparator sites. Information to inform Level 3 analysis will be gathered throughout the duration of the study, with extended site visits and post-discharge interviews at 30 and 90 days.

### Data management

Standardised templates will be provided to all participating sites with a request that quantitative data for Level 1 and Level 2 is provided in a format as close as possible to that template. A named member of staff at each site will link the different sources of data required at Level 2 (e.g. electronic files, paper-based notes) and supply the resulting information to the study team in a pseudo-anonymised format. Each set of data drawn from an individual patient’s notes will be recorded and linked by a unique, study-specific identification (ID) number, with identification keys held only by the relevant trust, to allow for source data verification as necessary. The qualitative data collected at Level 3 will be anonymised, with unique pseudonyms or identifiers assigned to each participating site or individual, and any identifiable information removed.

All data - including audio recordings, field notes and interview transcripts - will be stored at the University of Bristol, on a secure network drive that it is password protected, regularly backed-up and only accessible by members of the research team. Researchers will use University of Bristol-owned, password-protected laptops to store study information (e.g. typed field notes), while working at participating sites and only the researcher who collected the original data will be able to access information on their particular laptop. Interviews will be recorded using an encrypted voice recorder provided by the University of Bristol and transcription of these data will be conducted by a suitably qualified and approved transcription service.

The custodian of the study dataset will be the Chief Investigator (CI). The study database will be designed so as to protect patient information, in line with GCP guidelines and the Data Protection Act (DPA) 1998 (http://www.legislation.gov.uk/ukpga/1998/29/contents). All individuals recruited to the study will be identified only by a unique patient ID number on any study documentation. Furthermore, research staff will ensure that all participants’ anonymity is maintained through protective and secure handling / storage of information - both within their own research centre and at the participating sites. All documentation relating to the study will be accessible only to study staff and authorised personnel.

### Statistical justification for sample size

The sample size calculation was based on data from Level 2 sites which will be providing pseudo-anonymised details of all individual patient-level admissions over a 12-month period pre- and post-implementation of COPD care bundles. If we have 8 pairs of matched implementation and comparator sites in Level 2, this will provide a sample of around 10,000 admissions per year. Assuming an intra-cluster correlation co-efficient (ICC) of 0.01 and a cluster size of 625 - giving an effect size of 7.25 - there will be greater than 90 % power at the 5 % significance level to detect a 9 % absolute difference in the COPD re-admission rate at 28 days, assuming 30 % of patients are re-admitted in comparator sites.

A random sample of approximately one in five patients will be selected from Level 2 implementation and comparator sites for data on adherence to the care bundles and on delivery of the components of the care bundles. The total sample will be in the region of 2240 (16 × 140) cases. This will provide greater than 90 % power at 5 % significance to detect a difference in adherence to the care bundles from 30 to 70 %. In this case, the sample size has been increased according to a design effect of 29, corresponding to an ICC of 0.02 and a cluster size of 140.

### Data analysis

#### Quantitative analysis of effectiveness data

Level 1 data will be used to calculate the mean change following the introduction of care bundles for each site for all outcome measures. This mean change will be compared between implementer and comparator sites using ordinary linear regression, with adjustment for the following measures from the first period: number of COPD admissions, 28-day re-admission rate, and COPD mortality rate.

Level 2 data will be used in a series of appropriate regression models - depending on the outcome type - to compare the difference in change between the implementation and comparator groups. These models will include a ‘group x time-period’ interaction term to estimate the difference in change in outcome between the implementation and comparator sites before and after implementation of the care bundles. This approach will reflect the fact that the samples in the ‘before’ and ‘after’ periods will have captured data from sets of predominantly different individuals. All models will take appropriate account of the matched design by including indicator variables to distinguish each pair of sites. This will accommodate any between-site variation i.e. clustering, in outcomes. If it proves possible to identify patients who are admitted several times during the study period, a sensitivity analysis will be conducted with just the first of their admissions included, and the primary analysis elaborated if necessary to accommodate any correlation in outcome between a single patient’s episodes of care.

#### Quantitative analysis of cost-effectiveness data

The economic impact of care bundles at Level 1 sites will be evaluated by using trust-level data to describe the cost of COPD care per admission for both the implementation and comparator sites during the ‘before’ and ‘after’ time-periods. Since this analysis will use aggregated trust-level data, a simple unit-costing methodology will be deployed, based on a weighted average of non-elective COPD-related HRG codes. This will be used to estimate the incremental impact of bundles on COPD-related NHS secondary care costs.

A more detailed economic evaluation for the 90 days following the index admission will be undertaken in Level 2 sites. This will involve the estimation of per-patient secondary care NHS costs using a more in-depth HRG unit-costing methodology (www.ncbi.nlm.nih.gov/pubmed/21905152/) where patient-specific resource use will be valued using comprehensive, nationally representative sources e.g. NHS reference costs (https://www.gov.uk/government/publications/nhs-reference-costs-2014-to-2015/), the British National Formulary [21] (BNF) and Unit Costs of Health and Social Care (http://www.pssru.ac.uk/project-pages/unit-costs/2014/). Information on procedures and investigations undertaken during an in-patient stay e.g. x-rays, onward referrals, and drugs prescribed will be collected from a review of medical records and from routine data. The per-patient cost estimate will include the cost of admissions and re-admissions during the 90-day period. Linked information on 90-day mortality (including the number of days between discharge and death) will also be collected. The duration of the interactions between a sub-sample of admitted patients and clinical staff will be recorded in a ‘time and motion’ study at Level 3 sites in order to provide an estimate of the staff time involved in treating COPD patients, which will be costed using the sources described above and used to inform the Level 2 cost-effectiveness analysis.

This information on costs and mortality will allow an estimate of cost-effectiveness to be calculated as a ratio of the difference in NHS secondary care costs between intervention and comparator sites to the between-site differences in 90-day mortality. Uncertainty surrounding this estimate will be quantified using cost-effectiveness acceptability curves (CEACs). We will also use deterministic sensitivity analysis where appropriate.

Information on post-discharge resource use will also be collected from a sub-sample of consented Level 3 patients during telephone interviews. This information will be used to provide a descriptive analysis of differences between types of site in the use of primary care services, community care, and informal care up to 90 days post-discharge.

#### Qualitative analysis

The observational data will be collected in note form, developed as soon after the period of observation as possible and then word processed and uploaded into a proprietary qualitative analysis package e.g. NVivo [22]. All interviews will be digitally recorded, fully transcribed and anonymised and, similarly, will be uploaded and stored in readiness for coding and analysis. Both observational and interview data will then be examined using a cross-case thematic analysis [23]. This approach will be used to draw out the key issues in the data, using a coding framework which will be developed collaboratively by members of the research team. This process will enable both inductive and deductive analysis; focussing on the research questions, and also enabling the emergence of views and experiences expressed by interviewees. All data will be analysed and interpreted by at least two qualitative researchers, in order to cross-reference findings and ensure consistency and clarity. The analysis will seek to identify similarities and differences between sites, highlighting aspects that are likely to be transferable to other hospitals implementing – or intending to implement – care bundles.

### Ethical and regulatory issues

The study received full approval from South West (Frenchay) Research Ethics Committee on 12 September 2014. As a multi-centre research ethics committee, their single ethical opinion covers all aspects of the research and is valid across all participating sites. At the time of writing, the following sites have agreed to participate:

- Southmead Hospital, Bristol
- James Paget Hospital, Great Yarmouth
- Manchester Royal Infirmary, Manchester
- Worcestershire Royal Hospital, Worcester
- Heartlands Hospital, Birmingham
- Gloucestershire Royal Hospital, Gloucester
- Eastbourne District General Hospital, Eastbourne
- Lister Hospital, Stevenage
- Royal Bolton Hospital, Bolton
- New Cross Hospital, Wolverhampton
- Royal Surrey County Hospital, Guildford
- Bristol Royal Infirmary, Bristol
- Northwick Park Hospital, Harrow
- Leighton Hospital, Crewe
- Poole Hospital, Poole
- Royal Bournemouth General Hospital, Bournemouth
- Kings College Hospital, London
- Bedford Hospital, Bedford
- Peterborough City Hospital, Peterborough
- Southampton General Hospital, Southampton
- South Tyneside District Hospital, South Shields
- Stoke Mandeville Hospital, Aylesbury
- Prince Phillip Hospital, Llanelli
- Wexham Park Hospital, Slough
- Royal Albert Edward Infirmary, Wigan
- University Hospital Coventry, Coventry
- St Mary’s Hospital, Newport
- King George Hospital, Ilford
- Sandwell General Hospital, West Bromwich
- City Hospital, Birmingham
- Torbay District General Hospital, Torquay
- North Devon District Hospital, Barnstaple
- Princess of Wales’ Hospital, Bridgend
- Princess Alexandra Hospital, Harlow
- Luton and Dunstable University Hospital, Luton
- Epsom Hospital, Epsom

All necessary local research governance approvals will be obtained for each of the above-named sites prior to the start of data collection. The research will be conducted in accordance with the principles of the Declaration of Helsinki (http://www.wma.net/en/30publications/10policies/b3/), the principles of ICH good clinical practice (www.ich.org/) and in compliance with all other applicable regulatory requirements (www.hra.nhs.uk/resources/research-legislation-and-governance/research-governance-frameworks/). The study is registered on both the United Kingdom Clinical Research Network (UKCRN) Portfolio and the International Standard Randomised Controlled Trial Number (ISRCTN) registry.

## Discussion

Despite the fact that COPD is one of the most common causes of hospital admissions in the UK, there is no clear evidence as to how it can be most successfully managed either within or outside the acute setting, in order to improve in-patient care and reduce further re-admissions.

This study will use a controlled before-and-after design to evaluate the effectiveness of introducing standardised treatment packages i.e. care bundles, for patients with exacerbated COPD. Trust-level and patient-level data collected from a sample of hospitals across England and Wales will provide sufficient power to detect a meaningful difference in outcomes as regards the clinical effectiveness of COPD care bundles. In addition, a range of resource use data will inform a detailed analysis of their cost-effectiveness. Finally, the nested qualitative case-studies will offer insight into the processes underpinning care bundle implementation as well as the complex array of ‘real life’ factors implicated in looking after patients with this type of long-term respiratory illness.

Given the number of unplanned hospital admissions for this patient group and their rate of subsequent re-admission, it is hoped that the results from this evaluation will make an important and timely contribution to the existing evidence-base for the benefit of patients, clinicians, managers and policy-makers.

## Abbreviations

AECOPD: acute exacerbation of chronic obstructive pulmonary disease
BNF: British National Formulary
BTS: British Thoracic Society
CEAC: cost-effectiveness acceptability curve
CI: Chief Investigator
COPD: chronic obstructive pulmonary disease
CRN: clinical research network
DPA: Data Protection Act 1998
ED: emergency department
GCP: good clinical practice
GP: general practice or general practitioner
HRG: healthcare resource group
ICC: intra-cluster correlation co-efficient
ICD-10: International Statistical Classification of Diseases and Related Health Problems 10th Revision
ICH: International Conference on Harmonisation
ID: identification
IRAS: Integrated Research Application System
ISRCTN: International Standard Randomised Controlled Trial Number
NHS: National Health Service
NHSI: National Health Service Improvement
NIHR: National Institute for Health Research
NRES: National Research Ethics Service
OPCS: Office of Population, Census and Surveys
PI: Principal Investigator
R&D: Research & Development
REC: Research Ethics Committee
SD: standard deviation
SSI: site-specific information
UK: United Kingdom
UKCRN: United Kingdom Clinical Research Network
